# Prevention and Management of Malnutrition in Patients with Chronic Obstructive Pulmonary Disease: A Scoping Review

**DOI:** 10.3390/arm92050034

**Published:** 2024-09-06

**Authors:** Stefano Mancin, Sara Khadhraoui, Erica Starace, Simone Cosmai, Fabio Petrelli, Marco Sguanci, Giovanni Cangelosi, Beatrice Mazzoleni

**Affiliations:** 1 IRCCS Humanitas Research Hospital, Via Manzoni 56, 20089 Rozzano, Italy; stefano.mancin@humanitas.it (S.M.); erica.starace@humanitas.it (E.S.); 2 Department of Biomedical Sciences, Humanitas University, Via Rita Levi Montalcini 4, 20090 Pieve Emanuele, Italy; sarakhadhraoui1998@gmail.com (S.K.); simone.cosmai@hunimed.eu (S.C.); beatrice.mazzoleni@hunimed.eu (B.M.); 3 School of Pharmacy, Polo Medicina Sperimentale e Sanità Pubblica “Stefania Scuri”, Via Madonna delle Carceri 9, 62032 Camerino, Italy; 4 A.O. Polyclinic San Martino Hospital, Largo R. Benzi 10, 16132 Genova, Italy; sguancim@gmail.com; 5 Unit of Diabetology, ASUR Marche, 63900 Fermo, Italy; giovanni.cangelosi@virgilio.it

**Keywords:** chronic obstructive pulmonary disease, malnutrition, education, prevention, scoping review

## Abstract

**Highlights:**

**What are the main findings?**

- Chronic obstructive pulmonary disease represents one of the major causes of morbidity and mortality globally.
- Malnutrition is a common condition in patients with COPD, often requiring increased energy, protein, vitamin, and mineral requirements.

**What is the implication of the main finding?**

- Preventing malnutrition in COPD requires accurate screening with validated tools and personalized nutritional support.

**Abstract:**

Background: Chronic obstructive pulmonary disease (COPD) is linked to altered nutritional status due to increased catabolism, leading to muscle mass loss. This study aims to identify and map available evidence regarding multidisciplinary interventions focused on prevention, diagnosis and nutrition education, as well as the role of diet, to prevent and manage malnutrition in patients with COPD. Methods: A scoping review was conducted using the Cochrane, PubMed/Medline, CINAHL, Embase, Scopus, and Web of Science databases. This study adhered to the Arksey and O’Malley framework and JBI methodology. Results: Of the 1761 records identified, 15 were included. Evidence suggests that the Malnutrition Universal Screening Tool and Mini Nutritional Assessment are the most suitable screening scale. Guidelines have highlighted that personalized nutritional counseling is a very common intervention as it allows for a consideration of all physical, psychological, and social aspects of the patient. Conclusions: The role of healthcare professionals is crucial in the early identification of nutrition-related issues and in educating patients about the prevention and management of malnutrition, both in hospital and community settings. Key aspects include early malnutrition detection, personalized counseling and patient education, and a multidisciplinary approach. These findings provide a foundation for developing of targeted patient educational initiatives to improve the nutritional management of COPD patients.

## 1. Introduction

Chronic obstructive pulmonary disease (COPD) stands as a multifaceted lung condition marked by respiratory manifestations such as breathlessness, cough, and sputum production, arising from persistent and progressive airflow obstruction rooted in airway abnormalities like bronchitis or emphysema [1]. Its intricate genesis involves a convergence of genetic predisposition and environmental factors, spanning an individual’s lifespan and encompassing exposure to tobacco smoke, harmful particles, and gases from household and environmental pollution [2]. Beyond its immediate respiratory impact, COPD intricately intertwines with various chronic diseases, including vascular disorders, osteoporosis, muscle atrophy, and cancer [3], rendering its management and prevention a pivotal concern.

Recognizing the global magnitude of COPD, the guidelines from the European Respiratory Society (ERS) and the American Thoracic Society (ATS) underscore its profound impact on morbidity, mortality, and the substantial economic and social burdens it imposes. Despite its significance, COPD often evades detection until its clinically evident and moderately advanced stages, thereby leading to underestimations of its true societal impact [4]. Previous research [5] reveals a staggering prevalence of COPD in developing countries, affecting between 15 and 43 million individuals. In the United States, COPD ranks as the fourth leading cause of death, affecting 15.7 million Americans in 2018 [2]. Similarly, a recent study [6] sheds light on the shifting landscape of COPD prevalence, showing an increase among women in the European Union, while the incidence in men is decreasing [6]. However, COPD data collection faces challenges, marked by underestimations and limited awareness of the problem’s true extent. The accuracy of mortality statistics is hampered by uncertainties in the recording of COPD diagnoses and mortality data in health databases [1]. The surge in COPD-associated mortality, as highlighted by a guideline, is linked primarily to the expansion of smoking, aging populations, and the limited efficacy of available treatments [7]. Alarmingly, the World Health Organization (WHO) identifies COPD as a serious health issue. In 2019, it estimated COPD to be the third leading cause of death, surpassed only by ischemic heart disease and stroke [8]. Beyond its respiratory ramifications, COPD manifests as a condition characterized by nutritional imbalances, necessitating increased energy, protein, vitamin, and mineral intake. The gradual loss of muscle mass has severe implications on lung function and overall health, leading to a significant increase in disability and mortality [9]. Furthermore, this deterioration is closely associated with the widespread prevalence of malnutrition among patients with COPD, estimated at 30% according to a previous study [10], which indicates that malnutrition affects about one-third of hospitalized patients and one in five receiving outpatient treatment [11]. 

Identification of malnutrition risk is paramount, prompting the utilization of comprehensive assessment scales beyond the conventional body mass index (BMI). BMI alone may fall short in identifying all at-risk patients, necessitating routine and thorough screening during patient management and relevant clinical situations. Recognizing the pivotal role of a multidisciplinary team, preventive measures involve patient education encompassing dietary counseling, exercise promotion, breathing techniques, and stress management. Despite the existing guidelines, targeted educational programs are indispensable to enhancing malnutrition management in this patient population.

### Study Objective

The main objective of this scoping review was to identify and map available evidence regarding multidisciplinary interventions focused on prevention, diagnosis and nutritional counseling, as well as the role of diet, to prevent and manage malnutrition in patients with COPD.

## 2. Materials and Methods

### 2.1. Protocol and Registration

This scoping review followed a protocol registered prospectively on Open Science Framework on 4 December 2023 (https://doi.org/10.17605/OSF.IO/6FNPR, accessed 4 December 2023). In shaping the literature review, we adhered to the framework proposed by Arksey and O’Malley [12], incorporating the updated methodology endorsed by the Joanna Briggs Institute (JBI) [13]. Adherence to the Preferred Reporting Items for Systematic Reviews and Meta-Analyses extension for Scoping Reviews (PRISMA-ScR) [14] was maintained to enhance the study’s rigor. This comprehensive approach provides a solid foundation for systematically mapping the existing literature and ensuring transparency in reporting throughout the scoping review findings.

### 2.2. Formulation of Research Question

The formulation of the research question was guided by the PCC model [13], which includes three components: patient (P), concept (C), and context (C). Specifically, in this review, we included the following: P, patients with COPD; C, multidisciplinary interventions focused on diagnosis, prevention, and nutrition education, as well as the role of diet, to prevent and manage malnutrition; C, hospital or home setting.

### 2.3. Eligibility Criteria

Consistent with the established methodology [13,14], this review applied stringent eligibility criteria, following Arksey and O’Malley’s framework [12]; inclusion criteria underwent refinement through an initial exploration of databases such as PubMed/Medline and Google Scholar. This iterative process aimed to enhance confidence in the literature and ensure review accuracy. Inclusion criteria covered articles in English, and primary and secondary qualitative and quantitative studies related to COPD, associated malnutrition, diagnosis of malnutrition, or validated nutritional risk assessment scales. Exclusion criteria included expert opinion or consensus reports, irrelevant articles, duplicates, and reviews that did not provide fundamental information or evidence on the research problem. 

### 2.4. Information Sources

Aligned with the JBI framework [13,14], the identification of potentially relevant records involved a systematic literature search across four databases, Cochrane, PubMed/Medline, CINAHL, Embase, Scopus, and Web of Science databases, conducted in December 2023. All records considered potentially relevant were imported into EndNote 20 software (available at https://endnote.com/) [15]. Duplicate records were manually removed, ensuring an accurate compilation of the literature corpus for subsequent analysis.

### 2.5. Search Strategy

The development of search strings for this study involved utilizing keywords derived from the database thesaurus, including MeSH terms, along with additional keywords and Boolean operators. These search strings were tailored for the specific databases accessed. The selected keywords “undernutrition”, “diagnosis”, “COPD”, “education”, and “prevention and control” were chosen based on the study’s established eligibility criteria. To ensure a thorough exploration of the available literature, Google Scholar was consulted to retrieve potential additional records from the gray literature, adding depth and inclusiveness to the scoping review. Additionally, following the methodology [13,14], we planned to examine the references and citations of the full-text records obtained. This comprehensive approach not only reinforces the study’s rigor but also reflects the iterative nature of the scoping review process. For transparency and reproducibility, the detailed search strings employed in this review are provided in a Supplementary File.

### 2.6. Selection of Evidence Sources

The selection of evidence sources adhered to the guidelines outlined in the JBI methodology and the Arksey and O’Malley framework [12,13,14], ensuring a systematic and comprehensive approach. This process involved two stages, independently conducted by two researchers (SK and ES). Conflicts were resolved with the involvement of a third author (SM) who did not actively participate in the screening process. In the first stage, focused on titles and abstracts, articles related to non-COPD patients or with study designs inconsistent with the eligibility criteria were excluded. Lack of clarity on the population, intervention, comparison, or outcome also led to exclusion due to ambiguity. This phase efficiently excluded non-relevant publications, saving time in that we did not need to analyze their full texts. Moving to the next screening stage, full texts of records identified in the title and abstract screening were obtained through various approaches, including the dedicated EndNote function, internet research, and access to the journals where the studies were published. A comprehensive check of references and citations of these full texts was then conducted to identify additional eligible records. The final screening of full-text articles for eligibility was based on predefined inclusion and exclusion criteria, excluding incorrect publication types (e.g., books, chapters, congress contributions, and expert opinion or consensus reports), and those not addressing malnutrition in COPD adult patients. This process aimed to ensure the integrity of the evidence pool.

### 2.7. Data Charting Process

To facilitate data extraction for addressing research questions and achieving scoping review objectives, a data-charting form was developed, guided by JBI scoping review methodology [16]. Microsoft Excel and EndNote were employed for design and data extraction. The process was independently conducted by two researchers (SK and ES) to ensure a robust and unbiased approach. Any disparities or uncertainties in the extracted data were thoroughly discussed with a third author (SM) until a consensus was reached, aiming to enhance reliability and accuracy.

### 2.8. Data Extraction and Synthesis

Extracted data covered author information, publication year, country, study design, sample characteristics, research purpose, intervention, results, and limitations, systematically organized to address research objectives. The synthesis of results in this study followed a narrative approach based on the framework of Arksey and O’Malley [13]. The research categorized findings into educational strategies, screening interventions, and clinical treatments. Results are presented in a narrative format, supplemented by graphs and tables when relevant.

## 3. Results

**Figure 1:**
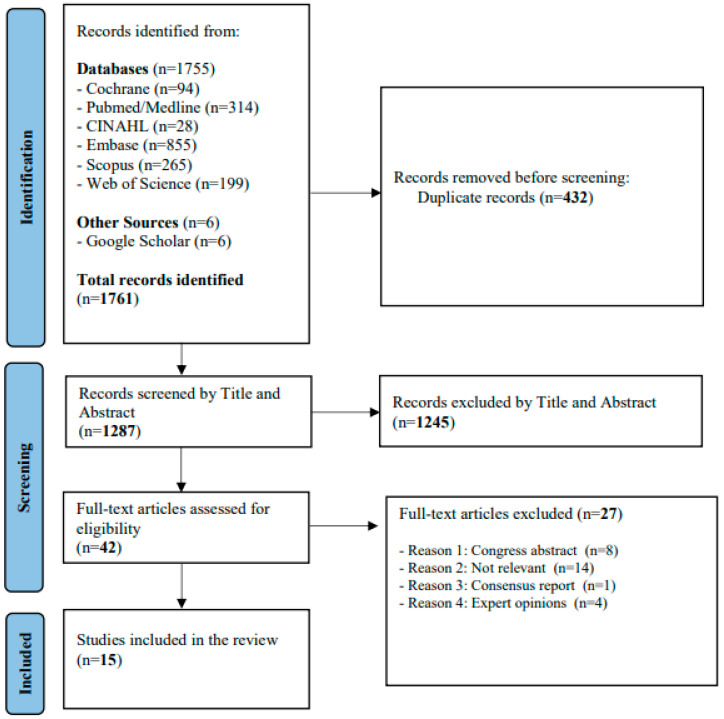
PRISMA-ScR flow chart of study selection process.

The initial search yielded 1761 results, of which 1755 were identified in primary and secondary literature databases, while 6 were identified through a gray literature search. After removing 432 duplicates, a total of 1329 records underwent screening, and 1287 were excluded after title and abstract review. Subsequently, 42 articles were evaluated after full-text reading, and 27 were excluded at this stage. Finally, 15 documents were considered relevant for inclusion in this scoping review. The selection process is illustrated in the PRISMA-ScR flowchart (Figure 1).

### 3.1. General Characteristics of the Studies Included

The articles included in this review consist of three guidelines [3,7,9], two literature reviews [17,18], three RCT [19,20,21], five cross sectional studies [22,23,24,25,26], one case–control study [27], and one qualitative study [28]. The studies focused on adult patients with COPD and involved patients from various countries, including the United Kingdom [9,18,21], the United States [3,7], Vietnam [20,22], Italy [17], Japan [19,25], the Netherlands [28], Turkey [23], Egypt [27], Taiwan [24], and Slovenia [26].

The primary studies included in this research described the effectiveness of personalized nutritional counseling in the treatment of malnutrition in patients with COPD [19]. Reviews and guidelines [3,7,9,17,18] provided a general perspective on COPD, illustrated the relationship between the disease and nutrition, discussed why patients with COPD suffer from malnutrition, and explored the role of healthcare professionals in the prevention and management of this complication [7,18,22,23,24,25,26]. The results of this research will be analyzed and divided into sections relating to the chronic disease and its relationship with nutritional status, screening interventions, malnutrition diagnosis, personalized individual consultancy, nutritional interventions, and the role of the multidisciplinary team in the treatment of malnutrition in COPD patients (Table 1).

### 3.2. Malnutrition in COPD Patients

**Figure 2:**
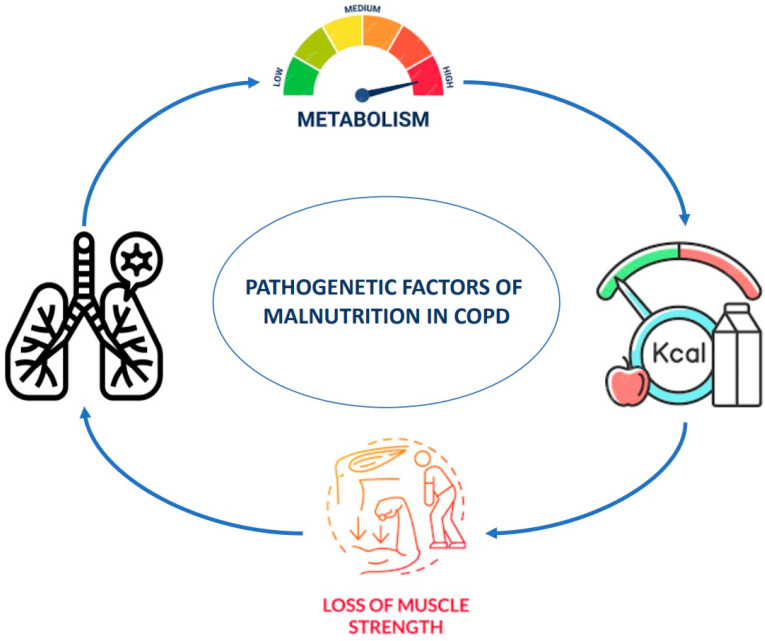
Pathophysiology of malnutrition in COPD. The figure illustrates the pathogenetic factors contributing to the development of malnutrition in patients with COPD.

Malnutrition in patients with COPD is associated with a range of issues, including compromised lung function, poor exercise tolerance, diminished quality of life, and increased mortality. Weight loss often occurs in these patients because their energy expenditure exceeds their energy intake, and factors such as reduced appetite, elevated levels of pro-inflammatory cytokines, and leptin contribute to this situation [7]. The severity of airflow obstruction is linked to malnutrition, as the inefficiency in respiratory function increases daily energy requirements [7]. A previous review [18] emphasizes that nutritional deficiencies are common in COPD patients, and optimizing nutritional status in these patients can help delay the progression of the disease and reduce the risks of early morbidity and mortality. A graphic representation of the pathophysiology of malnutrition in people with COPD is provided in Figure 2.

### 3.3. Nutritional Risk Screening

The accurate assessment of malnutrition risk in COPD patients is crucial to optimizing clinical management. Malnutrition can adversely affect disease progression and treatment response, necessitating timely nutritional interventions to improve clinical outcomes. Currently, there are no screening tools specifically tailored for this condition; thus, commonly used ones include Malnutrition Universal Screening Tool (MUST), Nutritional Risk Screening 2002 (NRS-2002), Subjective Global Assessment (SGA), and Mini Nutritional Assessment (MNA).

In the United Kingdom, a common tool for nutritional screening is MUST, developed by the British Association of Parenteral and Enteral Nutrition (BAPEN) and recommended in the National Institute for Health and Care Excellence (NICE) guidelines [29]. Supporting this, additional research [9] recommends the consistent use of MUST to assess malnutrition in COPD patients, both initially and during changes in the clinical condition. This assessment should be complemented by the measurement of mid-upper arm circumference, considering physiological, social, psychological, and environmental factors influencing the patient’s eating capacity.

Another valuable tool for malnutrition identification is SGA, employed in the Vietnamese study [20]. Assessing weight, height, food intake, gastrointestinal symptoms, and functional capacity, SGA classifies patients as well nourished (SGA-A), mildly to moderately malnourished (SGA-B), or severely malnourished (SGA-C). Despite its need for training and time, SGA proves reliable in diagnosing malnutrition. Furthermore, this study [20] mentioned Nutritional Risk Screening 2002 (NRS-2002) as a hospital tool to assess the risk of malnutrition. This scale considers criteria such as BMI, weight loss, reduced food intake, disease severity, and patient age, providing a more comprehensive assessment than MUST. The same authors emphasize that nutrition monitoring should not solely rely on body weight and weight loss, as they may lack sensitivity in detecting changes in nutritional status. Indicators like loss of appetite and reduced food intake can serve as early risk indicators.

**Figure 3:**
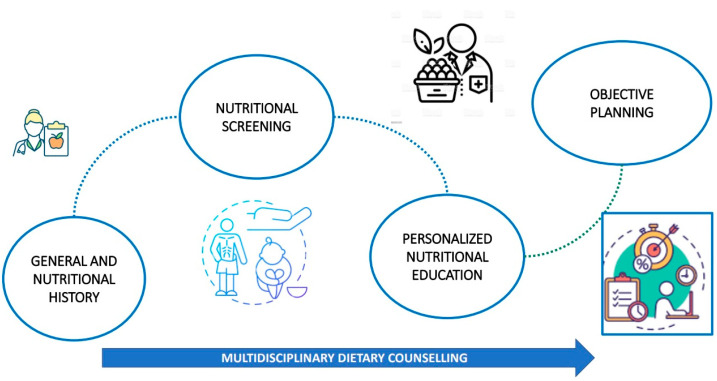
Multidisciplinary dietary counselling. the figure depicts the stages of multidisciplinary dietary treatment in patients with COPD.

Finally, four studies investigated nutritional risk using the MNA tool in COPD patients [23,24,25,26]. In a cross-sectional study of 105 COPD patients, a prevalence of malnutrition risk of 17% was observed, correlating lower spirometric parameters with low BMI and malnutrition, and correlating an increase in malnutrition with higher dyspnea scores (*p* = 0.002) [23]. Additionally, analyzing the correlation between disease severity, exercise capacity indicators, and malnutrition, a study on 83 stable COPD patients showed a strong correlation (*p* < 0.001) [24]. Lastly, in a prospective study of 108 COPD patients, 55% were at risk of malnutrition, with malnourished patients exhibiting higher rehospitalization rates (HR 2.93, 95% CI 1.05–7.32) [26]. These findings were confirmed by another study conducted on 60 COPD patients, where lower MNA scores were associated with a higher frequency of exacerbations (*p* = 0.019) [25] (Figure 3).

### 3.4. Individualized Counseling and Nutritional Education

A qualitative study [27] has highlighted the negative impacts of malnutrition on clinical outcomes and quality of life in COPD patients. Symptoms such as dry mouth and abdominal pain contribute to reduced appetite and food intake. Additionally, motivational strategies identified in a qualitative study have revealed themes such as “striving for a healthy lifestyle and diet”, “living independently”, and “a sense of continuity and duty”. Dietary counseling, incorporating a detailed history and personalized goal setting, addressed challenges in daily activities. However, despite the motivation to carry out healthy habits, only a minority of COPD patients met the criteria for a healthy diet [20]. In another context, an RCT conducted by Mouri et al. [19] investigated the eating behavior of 22 male outpatient COPD patients. Designed to control for gender effects on eating behavior, the study randomly assigned participants to either an educational intervention group or a regular care control group, ensuring an equal distribution of patients receiving home oxygen therapy. The intervention included a structured program supporting eating behavior, addressing influencing factors, and providing and instructing skills for adopting and maintaining healthy eating behavior. Results from the nutritional education programs revealed significant enhancements in meal adequacy and increased energy intake in the intervention group. Simultaneously, Weekes et al. [21] studied 59 malnourished patients, dividing them into an intervention group (n = 31) and a control group (n = 28). The intervention group received personalized dietary counseling, while the control group received only a nutritional pamphlet. Over one year, the study assessed parameters such as nutritional status, respiratory and musculoskeletal strength, lung function, dyspnea during activity, activities of daily living (ADL), and quality of life using St. George’s Respiratory Questionnaire (SGRQ). Results showed that the intervention group had increased calorie and protein intake, gaining weight during both the intervention and follow-up. The control group continued losing weight throughout. Significant differences were noted in total SGRQ scores (*p* = 0.02), Short Form-36 health change scores (*p* = 0.02), and dyspnea scores by the Medical Research Council (*p* = 0.03). The ADL score difference approached significance (*p* = 0.06). Dyspnea improved during the intervention but worsened in the subsequent 6 months. No differences were found in respiratory function or the strength of skeletal and respiratory muscles, and dyspnea during physical activity improved during the intervention period but worsened in the subsequent 6 months [21]. The authors demonstrated that personalized dietary advice resulted in weight gain and improved quality of life in 59 patients, with long-term benefits extending beyond the intervention period. In an RCT [20] involving 120 participants, divided into an intervention group (IG) receiving monthly personalized nutritional counseling using COPD-specific materials and a control group (CG) receiving the same materials without discussion, prevalent malnutrition was revealed among outpatient COPD patients. The intervention group, receiving personalized nutritional counseling, showed increased meal intake, variety in high-energy foods, and significant improvements in energy and protein intake, reducing weight loss rates and increasing body weight by 1.3 kg. Nutritional status significantly improved in the intervention group but worsened in the control group [21].

### 3.5. Role of Diet and Multidisciplinary Team in COPD

Proper nutrition is crucial in managing chronic respiratory diseases, impacting both nutritional status and the prevention of hypercapnia, maintaining low carbon dioxide levels [17]. A literature review proposed a COPD-specific dietary pyramid, advocating a low-carbohydrate diet to mitigate CO_2_ production, higher polyunsaturated fatty acid intake for anti-inflammatory effects, and elevated docosahexaenoic acid (DHA) levels linked to reduced COPD risk [17]. The approach emphasized a well-represented lipid profile, with omega-3 fatty acids aiding in preventing and treating chronic inflammation. Adequate protein intake, crucial for preventing muscle loss, was recommended at a 1–1.2 g/kg/body weight for prevention and at a 1.5 g/kg/body weight for treatment, including specific leucine intake at 2.5–2.8 g. Fiber incorporation, aiming for at least 25 g per day from whole grains, was underscored [18]. Consistent antioxidant intake was stressed, particularly through extra virgin olive oil (2–3 servings of 10 mL per day) and 30 g of nuts per day. The daily intake also included five servings of fruits and vegetables [17].

Two studies [3,17] highlighted the importance of vitamin D supplementation for COPD patients, as deficiency could lead to worsened lung function or osteoporosis. Hanson et al. [3] suggested regular monitoring of blood levels of 25-OH vitamin D for adult COPD patients. This study revealed positive associations between serum levels of 25(OH)D and lung function in 60% of the reviewed research. Additionally, vitamin D supplementation reduced exacerbations in COPD patients with levels below 25 nmol/L, considering the heightened risk of deficiency in COPD individuals [3,17]. Per the “Global Initiative for Chronic Obstructive Lung Disease” guideline [7], nutritional supplementation demonstrated significant improvements in the 6 min walk test, respiratory muscle strength, and overall health status in malnourished patients.

Addressing nutritional depletion in COPD individuals, Agustí et al. [7] stressed optimizing lung function, regular physical exercise, and tissue oxygenation improvement. A multidisciplinary approach, encompassing rehabilitation, nutritional support, and protein supplementation, demonstrated enhancements in fat mass, BMI, and exercise performance. Positive effects on mortality, body weight, mobility, and nutritional biomarkers were noted with protein supplementation in malnourished COPD patients post-hospitalization [7].

The included studies also highlight the need for the use of multidisciplinary teams in managing COPD.

Nurses, for example, are encouraged to promote patient participation in exercise programs as part of pulmonary rehabilitation to increase appetite and stimulate an anabolic response [18]. Also, in the “Management of Malnutrition in COPD” guidelines [7], the authors recommend the setting of personalized goals by healthcare professionals for malnourished COPD patients, including goals such as weight gain, muscle mass gain or, minimizing loss during flare-ups. All patients should receive basic education from the multidisciplinary team about COPD, its treatment, and the strategies to minimize dyspnea.

Regular reviews should evaluate changes in weight, grip strength, daily activities, physical appearance, taste and appetite.

## 4. Discussion

This scoping review examined evidence regarding the relationship between COPD and nutritional status, focusing on nutritional risk screening tools, the diagnosis of malnutrition using GLIM criteria, and the role of healthcare professionals in assessing and managing nutritional status. The prevalence of malnutrition associated with COPD, identified in various studies, underscores the importance of timely identification of nutritional problems in effectively managing this condition [11]. The use of validated screening tools such as MUST, MNA, and others like SGA and NRS-2002, enables comprehensive assessment in both outpatient and inpatient settings. However, further investigation is needed to assess the effectiveness of these tools in outpatient settings for COPD patients [20]. Internationally endorsed for diagnosing malnutrition not only in COPD but also in other diseases, especially chronic ones [30], these internationally validated criteria not only aim to overcome the limitations of previous diagnostic tools but also provide a standardized and internationally uniform instrument for diagnosing malnutrition.

Examining the impact of nutritional counseling on COPD patients, several studies [19,20,21,28] exploring both nutritional and functional dimensions have demonstrated potential benefits in weight gain and functional improvement, surpassing standard approaches and informational pamphlets. Long-term positive changes in dietary habits indicate a potential advantage over traditional ONS. Moreover, as demonstrated in various studies [20,21,28], it is clear that deeper patient involvement and understanding of diet are essential to promoting desirable eating behaviors. While short-term personalized nutritional counseling shows positive outcomes, the lack of long-term data underscores the need for further research to assess the lasting impact of such interventions. Addressing malnutrition at all stages of COPD is fundamental for respiratory function, body weight, and comorbidity management. Despite the uncertainty regarding the specific influence of macronutrient composition in outpatient COPD patients, adopting personalized dietary strategies, as for other chronic respiratory diseases [31], including energy- and protein-rich snacks and diet diversification, may prove to be a valid approach to prevent weight loss and improve overall quality of life. As recently demonstrated, a nutritional supplementation with antioxidants led to improvements in antioxidant deficiencies, quadricep strength, and total serum proteins, with no additional benefits to quadricep endurance, as supported by a recent double-blind randomized placebo-controlled trial [32].

Having nutritional skills and specific nutritional training [33,34] is crucial for healthcare professionals to ensure the success of such interventions, in order to encourage active collaboration. Although some limitations have been identified in the primary data, such as small samples sizes in individual studies and the use of subjective questionnaires, primarily employed in nutritional and quality of life assessments, this scoping review provides a valuable overview for healthcare professionals and researchers, serving as a basis for future studies and programs aimed at improving the nutritional management of COPD patients.

### Limits and Strengths

This review has some limitations, particularly the absence of large-scale studies and longitudinal evaluations within the cohort of COPD patients. Furthermore, the inclusion of gray literature sources and expert opinions could introduce potential biases, thus decreasing the robustness of the derived evidence. Nonetheless, this study makes significant contributions, such as the development of a nutritional screening pathway and support for personalized counseling as integral components in both the prevention and management of malnutrition among COPD patients.

## 5. Conclusions

The nutritional management of patients with COPD requires interdisciplinary collaboration involving doctors, nurses, dieticians, physiotherapists, pulmonologists, and other professionals, as supported by the studies included in this review. Since knowledge about nutrition and COPD management is continuously evolving, it is essential for healthcare professionals to stay updated and that patient nutrition education is delivered as effectively as possible.

This study aimed to identify and map multidisciplinary interventions focusing on prevention, diagnosis, education, and nutritional counseling, as well as the role of diet, to prevent and manage malnutrition in patients with COPD, laying the foundation for the development of future research in such a complex clinical field.

This review also sought to draw attention to the importance of developing a nutritional screening pathway and support for personalized counseling, highlighting how these elements are integral components in both the prevention and management of malnutrition in patients with COPD, opening up a modern field of research for a greater understanding of the clinical pathways involved.

## Figures and Tables

**Table 1:** General characteristics of the studies included.

Author, (Year)	Country	Research Design	Sample	Research Purpose	Intervention	Results	Limitations
Agustí et al. (2023) [7]	United States	Guideline	Patients with respiratory pathologies (*)	Nutritional assessment and management	Assessment and management of respiratory pathologies (Including nutrition)	Reduction in disease-related symptoms and in exacerbation risk	Insufficiently studied nutritional aspect
Ruby D. (2021) [27]	Egypt	Case-control study	COPD patients (Total n = 180; IG = 100; CG = 80)	Nutritional assessment and management	Nutritional assessment using MNA	90.6% of malnourished patients had dyspnea grade 4	N.A.
Anderson et al. (2020) [9]	United Kingdom	Guideline	COPD outpatients (*)	Nutritional assessment and management	Nutritional Screening Nutritional Therapy Patient Education	Management of nutritional assessment (healthcare professionals)	Insufficiently studied nutritional aspect
Hanson et al. (2020) [3]	United States	Guideline	COPD outpatients (*)	Nutritional assessment and management	Pharmacological and non-pharmacological treatment Management of malnutrition	Effectiveness of drug therapy BMI range 25–29.99 reduces the risk of mortality. Vitamin D reduces COPD exacerbations Importance of nutritional screening	Variability of results
Rondanelli et al. (2020) [17]	Italy	Narrative review	COPD outpatients (*)	Nutritional assessment and management	Implementation nutritional Pyramid for Preventing and Treating COPD malnutrition	Adequate energy intake Reduction of hypercapnia Reduction in chronic inflammation and oxidative Stress	Low number of high-quality studies
Mouri et al. (2020) [19]	Japan	RCT	COPD outpatients (male) (Total n = 22 IG = 11; CG = 11)	Personalized dietary counseling	Dietary Behavior Support Program	Increase in meal adequacy and energy intake	Sample and subjective evaluation tool
Nguyen et al. (2019) [20]	Vietnam	Cross-sectional study	COPD outpatients (n = 168)	Assessment of nutritional status	Nutritional assessment using SGA B/C	75% of the participants identified as malnourished	Sample Outcome measures Lost to follow-up
Nguyen et al. (2019) [22]	Vietnam	RCT	COPD outpatients (Total n = 120 IG = 60; CG = 60)	Personalized nutritional counseling	Assessing dietary intake	Increased energy and protein intake, body weight, nutritional status, muscle strength, and quality of life in the IG	Sample Outcome measures Dietary intake Lost to follow-up
Mete et al. (2018) [23]	Turkey	Cross-sectional study	COPD patients (n = 105)	Nutritional assessment and management	Nutritional assessment using MNA	Malnutrition risk associated with lower spirometric parameters (*p* = 0.002)	N.A.
Ter Beek et al. (2018) [28]	the Netherlands	Qualitative study	COPD outpatients (n = 12; ≥40 years)	Education	Nutritional interviews (30–45 min)	Key themes: “Striving to be as healthy as possible”, “maintaining independence”, and “promoting a sense of continuity and duty”	Non-generalizable results Lost to follow-up
Hsu MF et al. (2014) [24]	Taiwan	Cross-sectional study	COPD outpatients (n = 83)	Nutritional assessment and management	Nutritional assessment using MNA	MNA scores decreased with increasing disease severity, anthropometric parameters, and oxygen saturation e (all *p* < 0.01)	N.A.
Yoshikawa et al. (2014) [25]	Japan	Cross-sectional study	COPD outpatients (n = 60)	Nutritional assessment and management	Nutritional assessment using MNA Association between MNA and COPD exacerbation	MNA score associated with COPD exacerbation (*p*= 0.019)	Sample size
Benedik et al. (2011) [26]	Slovenia	Cross-sectional study	COPD patients (Total = 130; IG = 108; CG = 22)	Nutritional assessment and management	Nutritional assessment using MNA	MNA score decreased over GOLD stage (*p* = 0.02)	Sample size
Shepherd A. (2010) [18]	United Kingdom	Narrative review	COPD outpatients (*; underweight)	Nutritional assessment and management	Nutritional support and correction	Ensure an adequate nutritional status	N.A.
Weekes et. al. (2009) [21]	United Kingdom	RCT	COPD outpatients (Total n = 59 IG = 31; CG = 28)	Personalized dietary counseling	Individualized dietary counseling Health assessment with SGRQ questionnaire	Increased energy and protein intake Weight gain Improved SGRQ total score	Potential bias arising from inadequacy of blinding in treatment assignment and outcome assessment

Legend. Outpatients = no hospital setting; COPD = chronic obstructive pulmonary disease; BMI = body mass index; COPD = chronic obstructive pulmonary disease; CG = control group; GOLD = Global Initiative for Chronic Obstructive Lung Disease; SGRQ = St. George’s Respiratory Questionnaire (self-administered questionnaire designed to measure health deterioration in patients with airway diseases such as asthma and COPD); IG = intervention group; MUST = Malnutrition Universal Screening Tool; MNA = Mini Nutritional Assessment; N.A. = not applicable; RCT = randomized controlled trial; RHW = respiratory health workers; SGA = Subjective Global Assessment; * = sample size not indicated.

## Supplementary Materials

The following supporting information can be downloaded at: https://www.mdpi.com/article/10.3390/arm92050034/s1, Table S1: Search strategy; Table S2: PRISMA-ScR checklist.
